# Characterizing the University of California’s tenure-track teaching position from the faculty and administrator perspectives

**DOI:** 10.1371/journal.pone.0227633

**Published:** 2020-01-13

**Authors:** Ashley Harlow, Stanley M. Lo, Kem Saichaie, Brian K. Sato

**Affiliations:** 1 School of Education, University of California Irvine, Irvine, California, United States of America; 2 Section of Cell and Developmental Biology, Division of Biological Sciences and Program in Mathematics and Science Education, University of California San Diego, San Diego, California, United States of America; 3 Center for Educational Effectiveness, University of California Davis, Davis, California, United States of America; 4 Department of Molecular Biology & Biochemistry, University of California Irvine, Irvine, California, United States of America; Universidade de Mogi das Cruzes, BRAZIL

## Abstract

Teaching faculty are a potential mechanism to generate positive change in undergraduate STEM education. One such type of faculty is the Lecturer with Potential Security of Employment (L(P)SOE), a tenure-track faculty line within the University of California (UC) system. As a foundation for future studies, we sought to characterize individuals in the L(P)SOE position in terms of their background training, job expectations, and resources available for their success. Data were collected through an online survey completed by over 80% of STEM L(P)SOEs across the UC system, as well as interviews with over 20 deans and chairs in STEM departments at three UC campuses. From this work, we found that the majority of current L(P)SOEs were formally trained within their disciplines and not in an education field; however, they possessed substantial education experience, such as classroom teaching or participation in professional development opportunities. Expectations for time spent on teaching, research, and service are aligned between individuals within varying ranks of the L(P)SOE faculty and between L(P)SOEs and administrators. L(P)SOEs and administrators are also in agreement about what constitutes acceptable professional development activities. Interestingly, we identified differences that may reflect changes in the position over time, including increased start-up funds for more recently hired L(P)SOE faculty and a differing perspective on the role of discipline-based education research and scholarly activities between non-tenured and more senior L(P)SOEs. Overall, these data provide a snapshot of the L(P)SOE position that will aid in future work to identify the potential institutional impact of these individuals.

## Introduction

Improving undergraduate education in science, technology, engineering, and mathematics (STEM) is a topic of national conversation [1–15], as more than upwards one million STEM-educated graduates will be needed in the next decade to meet professional demands [15]. Recent research has provided significant evidence that increasing student engagement can result in improved learning outcomes, especially for underrepresented populations [16–18], and the question of how to implement these evidence-based instructional practices on an institutional scale is a major focus of STEM educators and researchers [19–23]. Possible means include developing curriculum and pedagogy, encouraging instructors to reflect on their instructional practices, and altering the institutional environment and fostering communities to promote instructional change [22]. One of these mechanisms for change includes revisiting instructional roles, namely who are the individuals in these positions and what expectations are for such positions, especially given decreasing numbers of full-time and tenure-track faculty hired at institutions in the United States [24].

In this paper, we review the recent trajectory of those who hold instructional roles, specifically focusing on the University of California’s (UC) Lecturer with Potential Security of Employment (L(P)SOE) position. The L(P)SOE is unique teaching-focused faculty position, as they are eligible for tenure and found only at research-focused institutions. Unlike other teaching-focused faculty lines, which encompass a number of different formal positions, L(P)SOEs all fall within a single job title. L(P)SOE faculty are of particular of interest now, as the position has appeared to change over recent years; based on alterations to job postings for L(P)SOE positions and informal conversations the authors have had with UC faculty, there seems to be an increasing emphasis on contributions beyond the classroom such as in discipline-based education research (DBER) and the scholarship of teaching and learning (SoTL).

## Background

An impediment to the transformation of undergraduate STEM education is the lack of time and incentive for tenure-track research faculty (which this work defines as tenure-track faculty who are evaluated primarily on the success of their research programs) to invest in their teaching [24]. One potential solution to this issue is creating and utilizing academic positions beyond the tenure-track research faculty, such as more teaching-focused faculty. Teaching faculty, in contrast to their tenure-track research colleagues, spend more of their time on classroom instruction and may be expected to be knowledgeable about evidence-based instructional practices. Such individuals could potentially have positive impacts both on students in their classrooms as well as their faculty colleagues [25].

The most common class of teaching faculty in higher education is the part-time or full-time non-tenure track lecturer [26–30], which we will refer to as “lecturer” in this paper. Between 1975 and 1995, the number of lecturers in the US increased by nearly 100% while the number of tenure-track research faculty decreased by 12% [31]. By 2016, nearly 70% of higher education instructors (excluding graduate students) were lecturers [32]. Lecturers are the predominant category of instructors in all levels of higher education institutions, including community colleges and both teaching-focused and research-focused four-year institutions [32]. There are a variety of reasons for the heavy reliance on lecturers, including decreased costs and the increased institutional flexibility associated with not providing a faculty member with tenure [33–34]. While lecturers are a prominent component of the higher education system, it is still unclear as to their impact on student outcomes. Ehrenberg and Zhang used national higher education data and found that increased hiring of non-tenure-track faculty led to decreased graduation rates, particularly at public, teaching-focused institutions [35]. Similarly, Jaeger and Eagan found that instruction from non-tenure-track faculty negatively impacts student persistence rates relative to their peers who are instructed only by tenure-track faculty [36]. However, Hoffmann and Oreopoulos examined student outcomes at a Canadian university and found that the type of instructor had little impact on grades and enrollment in subsequent courses in the discipline [37]. Further in contrast, Figlio et al. found that when students took first-year, first-semester courses with lecturers at a research-intensive university, they were likely to take more courses within that discipline and earn higher grades in subsequent courses [28]. Overall, the impact of lecturers is unclear, possibly due to the heterogeneous nature of the position as well as the wide variety of institution-types they are employed.

Another group of faculty specific to the STEM disciplines, is the Science Faculty with Education Specialties (SFES) [25, 38–40]. SFES are self-designated or peer-designated individuals who “take on a specialized role within science education in their disciplines” [39] within both tenure-track and non-tenure-track faculty positions. While originally studied in the California State University (CSU) system [38–39], this population has also been examined on a national level [39]. Both in the CSUs and nationally, SFES numbers have increased dramatically, roughly a 10-fold increase in both contexts since the 1980s [38–39]. These individuals are involved in a wide-variety of professional activities, including teaching, science education research, science research, and training of K-12 educators [38]. Despite their emphasized role in the education mission of their institutions, SFES were trained primarily in discipline-specific research with roughly 20% also earning graduate degrees in science education or a related field [40]. As far as SFES impact, the primary data collected thus far are from the perspective of the SFES themselves with these individuals reporting their roles as pedagogical resources for department colleagues, instigators of curriculum reform, and cultivators of departmental change in the education arena [25].

A previously unstudied group of teaching-focused faculty is the L(P)SOE position within the UC system. The L(P)SOE position is unique compared to lecturers and SFES. Unlike lecturers, L(P)SOEs are eligible for tenure, and unlike SFES, which encompass a number of different formal positions, L(P)SOEs can be categorized within one job title. The L(P)SOE position mirrors the tenure-track research faculty position, as L(P)SOEs are eligible for the same ranks, with titles including Lecturer with Potential Security of Employment (LPSOE—Assistant Professor equivalent), Lecturer with Security of Employment (LSOE—Associate Professor equivalent), and Senior Lecturer (Professor equivalent). (Note: In this paper, we will refer to individuals across all ranks in this position as L(P)SOEs. When referring to specific ranks, we will use the abbreviations above.) To earn tenure, L(P)SOEs are similarly evaluated on the three domains of research, teaching, and service. As with SFES, L(P)SOEs are embedded within departments, interacting with their tenure-track research peers in various capacities.

As a starting point to explore the potential impact of L(P)SOE faculty, in this paper, we characterize the position and the individuals employed within it. Through this work, we describe L(P)SOEs and the L(P)SOE faculty line across the UC system, both from the perspective of L(P)SOEs as well as key administrators, such as STEM deans and chairs who were involved in L(P)SOE hiring. Specifically, the following research questions were explored in this work:

1. What are the demographic characteristics of STEM L(P)SOE faculty?
2. What professional training do STEM L(P)SOE faculty possess, and is this training in-line with administrator expectations for the position?
3. What are the professional responsibilities of L(P)SOE faculty, and is there alignment between the L(P)SOE and administrator perspectives?
4. What resources are provided for L(P)SOE faculty to promote their success?

## Materials and methods

The study is intended to better understand the L(P)SOE faculty line within the UC system, particularly in the context of STEM L(P)SOEs. We defined STEM according to the National Science Foundation definition, which broadly includes disciplines in biological sciences, computer and information science, engineering, geosciences, mathematics, physical sciences, and social, behavioral, and economic sciences.

### Participants

#### L(P)SOE surveys

Data were collected at seven of the nine undergraduate-serving campuses in the UC system. Two of the universities were excluded as they did not have STEM L(P)SOEs employed at the time of survey distribution. We identified L(P)SOEs across the UC system by contacting a combination of Academic Personnel offices, STEM teaching centers, centers for teaching and learning, and campus registrars, as well as using departmental websites and personal contacts of L(P)SOEs known to the study authors. Surveys were sent to participants by email in Fall 2017 with detailed information about the survey and the purpose of the study. The survey was released to 146 L(P)SOE faculty. The response rate was 81.7% (N = 121/146). All data were collected in accordance to the University of California Irvine’s Institutional Review Board, which approved this study (Protocol 2015–2499).

#### Administrator surveys

STEM deans, chairs, vice-chairs, and L(P)SOE search committee chairs were asked to complete a brief survey. For the remainder of the manuscript, we will refer to these individuals as “administrators”. Administrators were identified by L(P)SOE faculty at the three UC campuses with the most L(P)SOEs employed, specifically due to their connection with the L(P)SOE hiring or mentorship process. Thirty-seven administrators were contacted, with a response rate of 62.1% (N = 23/37).

### L(P)SOE survey development

The L(P)SOE survey presented here was part of a larger survey instrument. For this paper, we will focus on three components of the survey: demographic information, job expectations, and resources available. Construction of questions for each domain are described below, and most measures were revised based on previously published instruments. Once created, five L(P)SOEs across the UC system were sent the survey in order to provide feedback on the items. The survey was then revised and administered to all STEM L(P)SOEs as described below. This portion of the survey can be found in the supplemental materials.

#### Demographic information

Participants were asked basic demographic information including: campus employed, gender, ethnicity/race, rank, length of time in their position, and previous training. Participants were asked to identify their previous formal and informal training in both their respective disciplines and in the field of education. Formal discipline training relates to research within a STEM field and includes earning a Ph.D. or a Master’s degree and working as a postdoctoral scholar. Formal education training also included a graduate degree or postdoctoral experience in an education or discipline-based education field. We also asked about informal experiences, including K-12 or undergraduate teaching, teaching professional development, or education research professional development, among others.

Participants who were employed for six months or less were not asked to complete the remainder of the survey items, as their limited time in the position may not provide meaningfully responses about their experience as L(P)SOE faculty. Seven individuals completed only this truncated version of the survey.

#### Job expectations

This measure is intended to understand, from the L(P)SOE perspective, their job expectations. Participants were asked approximately what percentage of time was expected in the three domains of responsibilities: teaching, scholarship, and service. Participants were also asked to list what is considered a scholarly activity and what types of courses they were expected to teach. These questions were modified from previous work that also sought to understand teaching faculty expectations [40].

#### Resources available

This section of the survey was intended to understand what resources are available to L(P)SOE faculty, including resources to conduct scholarly activity and the amount of their start-up funds. These items were created after speaking with a sample of administrators and other L(P)SOEs who were interested in potential resources that L(P)SOEs are provided that support their job.

### Administrator survey development

The administrator survey was meant to mirror the L(P)SOE survey to identify similarities and differences between the administrator and L(P)SOE perspectives. This included items regarding expectations for previous training; the percentage of time that L(P)SOE faculty should spend in teaching, scholarship, and service; what scholarly activities are acceptable for L(P)SOE faculty to pursue; and what types of courses L(P)SOE faculty are expected to teach.

### Data analysis

Survey results are primarily reported as descriptive statistics. Where appropriate for each research question, we use a simple Ordinary Least Squares (OLS) regression analysis to determine group differences (e.g., between LPSOE and Senior Lecturer) for each outcome. For each regression table, the LPSOE group was used as the comparison. All regression tables that show significant differences are reported in S1–S4 Tables. The following is the regression equation:

$$Y_{j}=\beta_{0}+\beta_{1}{\left(LPSOE\right)}_{j}+\beta_{2}{\left(LSOE\right)}_{j}+\beta_{3}(Senior)$$

Y_j_ represents the various outcome measures used in the study. *LPSOE*, *LSOE*, and *Senior Lecturer* (“*Senior*” in the above equation) are the three categorical variables with binary values of 0 and 1 to determine the rank of the participant. A “1” in any of the variables indicates their faculty rank.

To compare the administrator and L(P)SOE responses, a two-sample t-test was used to assess any significant differences between the means of the two groups. A two-sample t-test was used due to the relatively small sample of administrators (N = 23). The minimal data set is publicly available and can be found here: https://doi.org/10.7910/DVN/VZI6ZB

## Results

### Research Question 1: What are the demographics of STEM L(P)SOE faculty?

The study survey was completed by 121 STEM L(P)SOE faculty (Table 1). Females composed 50.4% of the survey group and males 49.6%. Most of the survey respondents identified as White (75.7%) with the second highest group being Asian (8.7%). Additionally, 27.3% were first-generation college graduates (defined as individuals whose parents did not complete a four-year degree in the United States). By rank, the majority of the respondents have not yet earned tenure, LPSOE (57.1%), followed by LSOE (25.9%) and Senior Lecturer (16.9%). The disciplines of L(P)SOE faculty were concentrated in four areas: biological sciences (30.4%), engineering (19.6%), social sciences (19.6%), and chemistry (17.9%); and the remaining 12.5% are in other STEM disciplines (which includes computer science, mathematics, pharmaceutical sciences, and physics). A majority of L(P)SOE faculty are employed at three campuses, representing 28.0%, 24.6%, and 19.5% respectively or accounting for a total of 72.1%.

**Table 1:** Demographic L(P)SOE faculty data.

	Count	Percentages
**Gender**		
Female	57	50.4%
Male	56	49.6%
**Ethnicity/race**		
Asian	10	8.7%
Black	3	2.6%
Hispanic	4	3.5%
White	87	75.7%
Other	2	1.7%
Multi-ethnic	3	2.50%
**College graduate status**		
First Generation College Graduate	33	27.3%
**Faculty rank**		
LPSOE	64	57.1%
LSOE	29	25.9%
Senior Lecturer	19	17.0%
**Discipline (home department)**		
Biological Sciences	34	30.4%
Chemistry	20	17.9%
Engineering	22	19.6%
Social Science	22	19.6%
Other STEM	14	12.5%
**University of California campus**		
Campus 1	33	28.0%
Campus 2	29	24.6%
Campus 3	23	19.5%
Campus 4	12	10.20%
Campus 5	10	8.50%
Campus 6	9	7.60%
Campus 7	2	1.70%

Demographic information for all L(P)SOE faculty who responded to the survey (N = 121). For college graduate status, the options were first generation college graduate or non-first-generation college graduate. For discipline, “other STEM” encompasses computer science, mathematics, pharmaceutical science, and physics.

### Research Question 2: What professional training do STEM L(P)SOE faculty possess, and is this training in-line with administrator expectations for the position?

Table 2 shows the proportion of formal discipline and educational training for the entire sample and for each faculty rank. In regard to discipline training, the vast majority of L(P)SOE faculty (90.1%) have a Ph.D. in their discipline, with 43.8% of the sample completing postdoctoral training in their discipline as well. However, very few L(P)SOE faculty had formal education training. Of the 121 individuals surveyed, only one has postdoctoral training in an education field, two have an education Ph.D., and two have an education Master’s degree. However, L(P)SOE faculty possess other education-related experience. Nearly all had taught at the undergraduate (71.1%) or K-12 (19.0%) levels and had participated in some form of teaching professional development activity (81.0%). A much smaller fraction also participated in science education professional development activities (10.7%). When comparing professional training between pre-tenure LPSOEs versus tenured LSOEs or Senior Lecturers, there was no significant differences across the ranks (S1 Table).

**Table 2:** L(P)SOE faculty formal discipline and education training.

	LPSOE		LSOE		Senior Lecturer		L(P)SOE Overall		Administrator Expectations of L(P)SOE Hires	
	Percentage	Count	Percentage	Count	Percentage	Count	Percentage	Count	Percentage	Count
**Discipline Training**										
Post Doc	43.8%	28	44.8%	13	57.9%	11	46.4%	52	56.5%	13
PhD	95.3%	61	96.6%	28	89.5%	17	94.6%	106	100%	23
Master’s	1.6%	1	3.4%	1	10.5%	2	3.3%	4	0	0
**Education Research Training**										
Education Post Doc	1.6%	1	0	0	0	0	0.8%	1	8.7%	2
Education PhD	1.6%.	1	0	0	5.3%	1	1.7%	2	0	0
Education Master’s	4.7%	3	3.4%	1	5.3%	1	4.1%	5	0	0

Formal discipline-specific training and education research training for L(P)SOE faculty including postdoctoral training, Ph.D. or Master’s degree. Additionally, administrators from three UC campuses (N = 23) were surveyed regarding their expectations for formal training from their L(P)SOE job candidates.

When initially searching for potential candidates, administrators involved in hiring L(P)SOE faculty had expectations that aligned with these findings. All of the 23 administrators we surveyed stated that a Ph.D. within the discipline was an expectation, with over half of them (56.5%) stating that postdoctoral experience within the discipline was important as well. Of these, only two (8.7%) stated that a postdoctoral position in an education field was encouraged, although even these individuals highlighted that it was not a requirement for the position. Similarly, the overwhelming majority expected prior undergraduate teaching experience (87.0%) with a strong preference for teaching professional development activities (70.0%). A smaller number felt that science education-focused professional development was expected (34.8%).

### Research Question 3: What are the professional responsibilities of L(P)SOE faculty, and is there alignment between the L(P)SOE and administrator perspectives?

As mentioned previously, L(P)SOE faculty are expected to not only be instructors but to contribute to scholarship and service. Thus, we were curious as to whether their reported training aligned with the expectations of the position. We collected data on the fraction of time that L(P)SOEs thought they should be spending on responsibilities in each category. Overall, respondents felt that L(P)SOEs should be spending 66.0% of their time on teaching activities, 16.7% on scholarship, and 17.3% service (Table 3). Across the various L(P)SOE faculty ranks, the LPSOE faculty ranked these percentages at 65.5% for teaching, 18.6% for scholarly activity, and 15.9% for service, having the highest percentage time on scholarly activity across ranks. LSOEs responded with 65.5%, 14.1% and 20.4% respectively, having the highest percentage time on service across ranks. Senior Lecturers reported the highest percentage time on teaching at 70.0%, with 15.8% for scholarly activity, and 14.2% for service. The difference in expectations for time spent on scholarship between LPSOE and LSOE faculty was significant (p<0.05) (S2 Table). Administrator expectations were in-line with L(P)SOE perceptions. On average, these individuals felt that 68.8% of L(P)SOE time should be spent on teaching, 17.8% on scholarship, and 14.3% on service. It is worth noting that six of the 23 surveyed administrators stated that there was not a formalized percentage of time that L(P)SOE faculty were expected to spend in each category in their particular department or school, and that it was not possible for them to answer this question.

**Table 3:** L(P)SOE faculty expected percentage of time spent on scholarly activity, service, and teaching.

Faculty Rank	LPSOE (% Time)	LSOE (% Time)	Senior (% Time)	L(P)SOE Overall (% Time)	Administrator Expectations of L(P)SOEs (% Time)
Scholarly Activity	18.6 (± 9.4)	14.1 (± 6.4)	14.2 (± 10.3)	16.7 (± 9.4)	14.3 (± 1.6)
Service	15.9 (± 8.1)	20.4 (± 17.7)	15.8 (± 10.0)	17.3 (± 11.9)	17.8 (± 1.9)
Teaching	65.5 (± 12.8)	65.5 (± 16.7)	70 (± 13.5)	66 (± 14.5)	68.8 (± 2.6)
N	51	28	18	101	17

Expectations for the amount of time that L(P)SOE faculty and administrators perceived L(P)SOEs should be spending in each of the three domains, scholarly activity, service, and teaching. These values are reported as average percentages (combined out of 100%). The standard deviation for each value is presented in parenthesis.

Additionally, we examined which course types L(P)SOE faculty felt they were expected to teach within their department. A substantial number noted that L(P)SOEs should teach lecture courses (97.0%) and laboratory courses (62.0%), as well as both lower- (93.0%) and upper-division (86.0%) courses. Administrators agreed with these expectations (lecture– 100%, laboratory– 87.0%, lower division– 100%, upper division– 78.3%). A much smaller percentage of both L(P)SOEs and administrators reported that L(P)SOEs should teach graduate courses (L(P)SOE– 29.0%, administrators– 8.7%) and science education courses (L(P)SOE– 14.0%, administrators– 21.7%). Responses from L(P)SOEs and administrators in regard to whether L(P)SOE faculty should teach graduate courses were statistically different (p <0.01 by t-test).

In regard to the scholarly activity component, we were curious to see what L(P)SOE faculty perceive as acceptable means to fulfill this requirement (Table 4). The most common selections included discipline-based education research (DBER) (76.5%), development of novel undergraduate curricula (65.3%), working to improve departmental teaching practices (62.2%), and assessment of departmental or institutional teaching (50.0%). When breaking these responses down by L(P)SOE ranks, responses were similar between LPSOE, LSOE, and Senior Lecturer faculty. Still, Senior Lecturers were less likely to denote DBER as an acceptable scholarly activity compared to LPSOE faculty (p<0.01) and were more likely to report K-12 teacher professional development was acceptable compared (p<0.01) (S3 Table). Administrator expectations in this regard were also in alignment (Table 4). All 23 surveyed highlighted discipline-based education research, and many noted that development of undergraduate curricula (65.0%), improving departmental teaching practices (70.0%), and assessment of departmental or institutional teaching (52.2%) were acceptable scholarly activities. Administrators were nearly twice as likely to report that L(P)SOEs providing professional development opportunities for faculty or future faculty was considered to be an appropriate example of a scholarly activity (26.5% for L(P)SOEs versus 47.8% for administrators, p<0.05).

**Table 4:** L(P)SOE faculty perception of acceptable scholarly activities.

Activity	LPSOE (% Responses)	LSOE (% Responses)	Senior Lecturer (% Responses)	L(P)SOE Overall (% Responses)	Administrators (% responses)
Discipline-Based Education Research	82.0	81.5	50.0	76.5	100
Development of Undergraduate Curricula	64.0	63.0	77.8	65.3	65.0
Improving Departmental Teaching Practices	60.0	55.6	72.2	62.2	70.0
Assessment of Departmental or Institutional Teaching	50.0	48.2	50.0	50.0	52.2
Discipline-Based Research	46.0	33.3	55.6	45.9	39.1
Undergraduate Mentorship	40.0	55.6	33.3	42.9	43.5
Providing Faculty or Future Faculty Professional Development	28.0	25.9	27.8	26.5*	47.8*
Providing K-12 Teacher Professional Development	2.0	7.4	22.2	8.2	13.0
Development of K-12 Curricula	4.0	3.7	0.0	4.1	4.3

L(P)SOE and administrator perceptions of activities that are acceptable examples of scholarly activities for the L(P)SOE position. The percentage of survey respondents in each category is reported. Two-sampled t-tests were used to report mean differences between the L(P)SOE overall responses and the administrator responses.

* p<0.05

### Research Question 4: What resources are provided for L(P)SOE faculty to promote their success?

We surveyed L(P)SOE faculty regarding the resources provided by their departments, including the types of support available to pursue scholarly work and the amount of start-up funds. When L(P)SOE faculty were presented with the statement “I have the tools and resources to do my job well”, a majority of respondents strongly agreed (21.2%) or agreed (46.7%) with the statement while another 20.2% slightly agreed. The cumulative disagreement with this statement (slightly disagree, disagree, strongly disagree) was only 13.2%.

With regard to support for scholarly activities, the most common forms included funds specifically designated for conference attendance (41.3%), the opportunity for sabbatical (38.8%), and the ability to hire undergraduate student researchers (23.5%). Very few L(P)SOE faculty reported that they had the opportunity to obtain reduced teaching responsibilities (10.7%) or support to sponsor graduate student researchers (7.4%). We also asked about financial support in the form of start-up funds (Table 5). The most common amount provided for L(P)SOEs ranged between $10,000 to $20,000 (36.3%). Some respondents reported receiving no start-up funds (14.1%) when they were hired. This was particularly true for Senior Lecturers: Nearly 40% did not receive start-up funds, a proportion that was significantly higher than LPSOE faculty (p<0.001) (S4 Table).

**Table 5:** L(P)SOE faculty start-up fund amount.

Start-Up Fund Amount	LPSOE (% Responses)	LSOE (% Responses)	Senior (% Responses)	L(P)SOE Overall (% Responses)
No Start-Up Funds	3.9	18.5	38.9	14.1
Less than $10,000	17.7	3.7	16.7	13.1
$10,000 to $20,000	35.3	37.0	33.3	36.4
$21,000 to $30,000	9.8	22.2	0.0	12.1
$31,000 to $50,000	23.5	11.1	5.6	16.1
$51,000 to $100,000	6.1	9.8	3.7	0.0
Greater than $100,000	2.0	0.0	3.7	5.6

L(P)SOEs reported the amount of start-up funds they received when hired. The percentage of survey respondents who selected each range is reported.

## Discussion

In this study, we describe the L(P)SOE faculty line in the UC system, specifically in relation to their demographic characteristics, professional training, professional responsibilities, and the campus resources that are provided to support L(P)SOEs.

Comparison across L(P)SOE ranks and triangulation with data from the survey of administrators, who have been involved in the hiring and mentoring of L(P)SOEs, provide additional insights into the university and campus contexts for our research questions. Here, we highlight a few important patterns.

Demographically, L(P)SOE faculty are equally split in terms of gender, are predominantly white (about 75%) with close to 10% identifying as Asian, and over one-quarter are the first in their family to graduate college. Additionally, the majority (nearly 60%) are not yet tenured LPSOE faculty, and by discipline, approximately 30% are affiliated with the biological sciences, 20% engineering, 20% chemistry, and 20% social sciences. The distribution of L(P)SOE faculty is also not homogenous with three UC campuses hiring nearly 75% of the survey population. The gender and ethnicity data are interesting in light of nationwide faculty demographics. A recent study by Li and Koedel [41] examined faculty demographics of the top 40 public institutions according to *US News and World Report*, of which the majority of institutions from our study are included. Similar to our findings, STEM faculty are mostly white (76–83% depending on discipline) with Asian being the next most predominant. In contrast to our L(P)SOE sample, faculty in the Li and Koedel study are mostly male (70–80% with the exception being Sociology at 53% male), hinting that the hiring of females into L(P)SOE positions may be more acceptable to departments than into tenure-track research faculty positions for reasons that are beyond the scope of this study. Nonetheless, this trend can have significant implications for increasing equity in undergraduate STEM educadtion, as prior work has demonstrated the importance of instructor gender on female student participation and success in STEM fields [42–44].

We also found that there are high-levels of agreements between L(P)SOEs and administrators on professional training and responsibilities. Overall, the L(P)SOE faculty population is relatively homogenous in terms of professional training. Uniformly across all three L(P)SOE ranks, nearly all surveyed have a PhD in their discipline, and roughly half also have postdoctoral experience in their discipline, whereas very few possess formal training in an education field. These proportions are consistent with the data from the administrator survey. In terms of professional responsibilities, both L(P)SOEs and administrators indicated that about two-thirds of L(P)SOE time and effort should be focused on teaching, with the remaining one-third evenly divided across scholarly activity and service. These administrators are likely to represent the departmental and institutional cultures, norms, and values that led to the hiring of this current population of L(P)SOEs. Therefore, it is perhaps not surprising that the expected training (from administrators) matches the actual training of L(P)SOEs and that there is a consensus on the distribution of time or effort on teaching, scholarship, and service. The relative uniformity of L(P)SOE responsibilities is also in contrast to the more diverse expectations reported in the SFES positions [45]. This difference may be due to the L(P)SOE positions being under one faculty line or title within a single university system, whereas SFES are self-designated or peer-designated individuals in both tenure-track and non-tenure-track positions across many different institutions [39].

Across ranks, L(P)SOEs reported some differences in professional responsibilities, such as the specific types of work that would be considered scholarly activity. Compared to LPSOEs, Senior Lecturers are statistically less likely to indicate DBER as an acceptable form of scholarly activity and statistically more likely to indicate professional development for K-12 educators as scholarly activities. These results suggest a potential shift in the position in terms of the expectations for types of scholarly activities. LSOE faculty also reported a significantly lower expectation in time spent on scholarly activity relative to LPSOEs, further emphasizing the shifting expectations of the position over time. Consistent with this idea that more senior L(P)SOE faculty have lesser scholarly activity expectations, Senior Lecturers were statistically more likely to not receive start-up funds compared to LPSOEs.

One unique aspect of the L(P)SOE position relative to other teaching focused faculty is this expectation for scholarly activity. Despite the fact that respondents felt that about one-sixth of their time should focus on scholarly activities, many signs contradicted this expectation. For example, very few L(P)SOEs had formal training in any type of educational research. This is in contrast to the more recently hired LPSOEs recognizing the importance of DBER scholarship to their position. Therefore, it is important for administrators to acknowledge this challenge for L(P)SOEs, either by providing additional training in conducting DBER, for example through the establishment of workshops in DBER skills or mentorships with DBER scholars, or by hiring L(P)SOE candidates with this education research background. This is especially important for the success of L(P)SOE faculty; in the analogous SFES literature, a substantial portion of SFESs reported the desire to leave their positions because of the lack of support for their work in the departments [39–40, 25].

We highlighted some differences between L(P)SOE and SFES faculty in the previous paragraphs. However, there are also some similarities. First, in the existing SFES literature, SFESs reported that they have less teaching responsibilities in graduate courses compared to undergraduate education [39]. Similarly, in our current L(P)SOE study, only a small number of L(P)SOEs (under 30%) and administrators (under 10%) reported that graduate courses would be part of the L(P)SOE teaching portfolio. Second, providing professional development or being a resource for colleagues is one of the main activities described for SFESs [39, 25]. In our current L(P)SOE study, administrators were almost twice as likely compared to L(P)SOEs to report that providing professional development for faculty or future faculty as a form of scholarly activity, suggesting that they may have an additional implicit view of L(P)SOEs as potential resources in the departments for colleagues on undergraduate curriculum and instruction. Therefore, even though the L(P)SOE position is more uniform in expectations in terms of teaching, scholarship, and service than that of the diverse SFES positions, there are nonetheless some similarities in how administrators may implicitly understand these teaching-focused faculty positions.

Future work can focus on exploring the administrator perspective, so we can better understand how administrators view the L(P)SOE faculty line and the role of L(P)SOEs within their department. Such qualitative data will complement the current L(P)SOE study, which relies primarily on descriptive statistics, which limits the depth of the conclusions we can make. Interviewing administrators will provide further triangulation by adding qualitative data, so we can better understand if and how administrators perceive L(P)SOEs as potential resources and even change agents in the departments on undergraduate STEM education. With the tremendous push to improve STEM education nationwide, identifying an institutional structure, such as a teaching-focused faculty line like L(P)SOEs, to facilitate this transformation would be of immense interest to leaders in higher education. While it is yet to be seen whether the L(P)SOE model is an example of this, we believe this study is an important step in the direction to answering these questions.

## Conclusions

To our knowledge, this study is the first to characterize the teaching-focused L(P)SOE faculty line. The overall population of L(P)SOE faculty is limited, and even though we surveyed over 80% of the STEM L(P)SOE population in the UC system, the number of individuals in the Senior Lecturer rank is small, thus making comparisons across ranks challenging. Such comparisons are particularly important, as we were still able to observe some interesting difference across L(P)SOE ranks, suggesting potential shifts in professional responsibilities in the L(P)SOE faculty line over time. As the L(P)SOE population continues to grow and as LPSOEs and LSOEs progress through the tenure track, the number of Senior Lecturers will increase, thus leading to the need for future work exploring this unique position and rank in larger numbers. Nonetheless, this study provides a snapshot of individuals in the L(P)SOE faculty line at a particular time point. Repeating the survey in the future will also allow us to more firmly and clearly identify changes in the L(P)SOE faculty line over time.

## Supporting information

S1 Table Comparison of formal discipline and education training. Simple OLS regression was used to identify any significant differences between groups in regard to formal discipline and education training. “*–*” denotes comparison group. Standard error is in parentheses.
(DOCX)

S2 Table Comparison of expected percentage of time spent on scholarly activity, service and teaching by faculty rank. Simple OLS regression was used to identify any significant differences between groups in regard to expectations for the percentage of time spent on scholarly activity, service, and teaching. “*–*” denotes comparison group. Standard error is in parentheses. * p < .05.
(DOCX)

S3 Table Comparison of perceptions of acceptable scholarly activities. Simple OLS regression was used to identify any significant differences between groups in regard to perceptions of acceptable scholarly activities. “*–*” denotes comparison group. Standard error is in parentheses. **p < .01.
(DOCX)

S4 Table Comparison of faculty start-up package. Simple OLS regression was used to identify any significant differences between groups in regard to faculty start up package. “*–*” denotes comparison group. Standard error is in parentheses. ***p < .001.
(DOCX)
