# Supplementary material for: Characterizing the University of California’s tenure-track teaching position from the faculty and administrator perspectives
Source: PLoS One. 2020 Jan 13;15(1):e0227633. doi: 10.1371/journal.pone.0227633 (PMC6957150; doi:10.1371/journal.pone.0227633)
Supplement: S1 Table — Simple OLS regression was used to identify any significant differences between groups in regard to formal discipline and education training. “–” denotes comparison group. Standard error is in parentheses. (DOCX) [file pone.0227633.s001.docx]

**Table S1 Comparison of Formal Discipline and Education Training**

|  | Discipline Training | | | Education Training | | |
| --- | --- | --- | --- | --- | --- | --- |
|  | Postdoctoral Training | PhD | Master’s Degree | Postoctoral Training | PhD | Master's Degree |
| LPSOE | - | - | - | - | - | - |
|  | - | - | - | - | - | - |
| LSOE | 0.013 | -0.011 | 0.017 | -0.059 | -0.083 | 0.054 |
|  | (0.120) | (0.120) | (0.048) | (0.041) | (0.079) | (0.042) |
| Senior Lecturer | 0.124 | -0.196 | 0.092 | -0.059 | -0.046 | -0.020 |
|  | (0.138) | (0.138) | (0.055) | (0.048) | (0.092) | (0.048) |
| N | 96 | 96 | 96 | 96 | 96 | 96 |
| R-sq | 0.009 | 0.023 | 0.029 | 0.028 | 0.012 | 0.025 |

Simple OLS regression was used to identify any significant differences between groups in regard to formal discipline and education training. “*–*” denotes comparison group. Standard error is in parentheses.
