# Supplementary material for: Characterizing the University of California’s tenure-track teaching position from the faculty and administrator perspectives
Source: PLoS One. 2020 Jan 13;15(1):e0227633. doi: 10.1371/journal.pone.0227633 (PMC6957150; doi:10.1371/journal.pone.0227633)
Supplement: S2 Table — Simple OLS regression was used to identify any significant differences between groups in regard to expectations for the percentage of time spent on scholarly activity, service, and teaching. “–” denotes comparison group. Standard error is in parentheses. * p < .05. (DOCX) [file pone.0227633.s002.docx]

**Table S2 Comparison of Expected Percentage of Time Spent on Scholarly Activity, Service and Teaching by Faculty Rank**

|  | Scholarly Activity | Service | Teaching |
| --- | --- | --- | --- |
| LPSOE | - | - | - |
|  | - | - | - |
| LSOE | -4.573* | 3.952 | 0.621 |
|  | (2.113) | (2.842) | (3.364) |
| Senior Lecturer | -4.480 | -0.029 | 4.510 |
|  | (2.434) | (3.274) | (3.875) |
| N | 96 | 96 | 96 |
| R-sq | 0.063 | 0.022 | 0.015 |

Simple OLS regression was used to identify any significant differences between groups in regard to expectations for the percentage of time spent on scholarly activity, service, and teaching. “*–*” denotes comparison group. Standard error is in parentheses. * p<.05
