# Supplementary material for: Characterizing the University of California’s tenure-track teaching position from the faculty and administrator perspectives
Source: PLoS One. 2020 Jan 13;15(1):e0227633. doi: 10.1371/journal.pone.0227633 (PMC6957150; doi:10.1371/journal.pone.0227633)
Supplement: S3 Table — Simple OLS regression was used to identify any significant differences between groups in regard to perceptions of acceptable scholarly activities. “–” denotes comparison group. Standard error is in parentheses. **p < .01. (DOCX) [file pone.0227633.s003.docx]

**Table S3 Comparison of Perceptions of Acceptable Scholarly Activities**

|  | DBER | Development of Undergraduate Curricula | Improving Departmental Teaching Practices | Assessment of Departmental or Institutional Teaching | Discipline -Based Research | Undergraduate Mentorship | Providing Faculty or Future Faculty Professional Development | Providing K-12 Teacher Professional Development | Development of K-12 Curricula |
| --- | --- | --- | --- | --- | --- | --- | --- | --- | --- |
| LPSOE | - | - | - | - | - | - | - | - | - |
|  | - | - | - | - | - | - | - | - | - |
| LSOE | -0.005 | -0.010 | -0.044 | -0.019 | -0.127 | 0.156 | -0.021 | 0.054 | -0.003 |
|  | (0.099) | (0.114) | (0.118) | (0.121) | (0.119) | (0.119) | (0.108) | (0.061) | (0.042) |
| Senior Lecturer | -0.320** | 0.138 | 0.122 | 0.000 | 0.096 | -0.067 | -0.002 | 0.202** | -0.040 |
|  | (0.114) | (0.131) | (0.135) | (0.140) | (0.137) | (0.136) | (0.125) | (0.070) | (0.049) |
| N | 95 | 95 | 95 | 95 | 95 | 95 | 95 | 95 | 95 |
| R-sq | 0.085 | 0.014 | 0.014 | 0.000 | 0.024 | 0.027 | 0.000 | 0.083 | 0.008 |

Simple OLS regression was used to identify any significant differences between groups in regard to perceptions of acceptable scholarly activities. “*–*” denotes comparison group. Standard error is in parentheses. **p<.01
