# Supplementary material for: Characterizing the University of California’s tenure-track teaching position from the faculty and administrator perspectives
Source: PLoS One. 2020 Jan 13;15(1):e0227633. doi: 10.1371/journal.pone.0227633 (PMC6957150; doi:10.1371/journal.pone.0227633)
Supplement: S4 Table — Simple OLS regression was used to identify any significant differences between groups in regard to faculty start up package. “–” denotes comparison group. Standard error is in parentheses. ***p < .001. (DOCX) [file pone.0227633.s004.docx]

**Table S4 Comparison of Faculty Start-Up Package**

|  | No Start-Up | Less than 10K | 10-20K | 21-30K | 31-50K | 51-100K | Greater than 100K |
| --- | --- | --- | --- | --- | --- | --- | --- |
| LPSOE | - | - | - | - | - | - | - |
|  | - | - | - | - | - | - | - |
| LSOE | 0.146 | -0.139 | 0.017 | 0.124 | -0.124 | -0.061 | 0.037 |
|  | (0.079) | (0.081) | (0.116) | (0.075) | (0.088) | (0.058) | (0.034) |
| Senior Lecturer | 0.350*** | -0.010 | -0.020 | -0.098 | -0.180 | -0.098 | 0.056 |
|  | (0.091) | (0.094) | (0.133) | (0.086) | (0.102) | (0.067) | (0.039) |
| N | 96 | 96 | 96 | 96 | 96 | 96 | 96 |
| R-sq | 0.141 | 0.032 | 0.001 | 0.058 | 0.041 | 0.027 | 0.026 |

Simple OLS regression was used to identify any significant differences between groups in regard to faculty start up package. “*–*” denotes comparison group. Standard error is in parentheses. ***p<.001
